# Preservation of Auditory P300-Like Potentials in Cortical Deafness

**DOI:** 10.1371/journal.pone.0029909

**Published:** 2012-01-17

**Authors:** Marianna Cavinato, Jessica Rigon, Chiara Volpato, Carlo Semenza, Francesco Piccione

**Affiliations:** 1 San Camillo Foundation, Institute of Care and Research, Venice, Italy; 2 Department of Neuroscience, University of Padova, Padova, Italy; University of British Columbia, Canada

## Abstract

The phenomenon of blindsight has been largely studied and refers to residual abilities of blind patients without an acknowledged visual awareness. Similarly, “deaf hearing” might represent a further example of dissociation between detection and perception of sounds.

Here we report the rare case of a patient with a persistent and complete cortical deafness caused by damage to the bilateral temporo-parietal lobes who occasionally showed unexpected reactions to environmental sounds despite she denied hearing. We applied for the first time electrophysiological techniques to better understand auditory processing and perceptual awareness of the patient. While auditory brainstem responses were within normal limits, no middle- and long-latency waveforms could be identified. However, event-related potentials showed conflicting results. While the Mismatch Negativity could not be evoked, robust P3-like waveforms were surprisingly found in the latency range of 600–700 ms. The generation of P3-like potentials, despite extensive destruction of the auditory cortex, might imply the integrity of independent circuits necessary to process auditory stimuli even in the absence of consciousness of sound. Our results support the reverse hierarchy theory that asserts that the higher levels of the hierarchy are immediately available for perception, while low-level information requires more specific conditions. The accurate characterization in terms of anatomy and neurophysiology of the auditory lesions might facilitate understanding of the neural substrates involved in deaf-hearing.

## Introduction

Extensive bilateral lesions of the primary auditory cortex can produce a complete loss of hearing, known as cortical deafness. Bitemporal damage required to produce this deficit is rare and is most frequently caused by ischemic cerebrovascular accident [1], [2].

There is a relatively limited research on this condition, likely because cortical deafness is typically transient and rapidly resolves in more selective hearing disorders, such as auditory agnosia or word deafness [3]. In some isolated cases, however, cortical deafness may be permanent [4].

Generally, cortical deafness is characterized by the complete disruption of central auditory processing, despite intact peripheral auditory function [2]. However, from the limited literature available, several authors have described the presence of residual auditory behaviors in completely deaf patients [2], [5], [6]. Relatives reported occasional unexpected reactions of patients to environmental sounds despite they denied hearing. This phenomenon has been likened to blindsight, referring to residual visual abilities of individuals without acknowledged awareness [7]. Similarly, “deaf-hearing” seems to imply a dissociation between detection and perception of sounds.

In the present report, we had the opportunity to study a patient with severe persistent cortical deafness caused by two consecutive strokes. The lesions extended into both Heschl's gyri, insulae and superior temporal gyri. Surprisingly, the patient showed some reactions to unexpected sounds, despite the lack of any startle response to loud noises. We adopted an electrophysiological approach involving auditory evoked potentials (AEPs) and event related potentials (ERPs) to assess auditory processing and perceptual awareness. Generally, auditory potentials are characterized by a time-resolution on the order of milliseconds and can therefore provide temporal correlates of the stages of information processing between stimulus and response. AEPs reflect the activation of auditory nerve and central auditory pathways up to the primary auditory cortex [8]. Differently, ERP recordings represent a valuable method to provide quantitative information about the perception and higher order processing of acoustic stimuli [9], [10].

On the basis of these assumptions, the present study should provide a procedure by which electrophysiological methods can be used to study auditory unawareness or deaf-hearing.

## Methods

### Ethics statement

The present study was approved by the Research Ethics Committee (REC) of the Scientific Foundation “San Camillo” and was compliant with the declaration of Helsinki guidelines. Written informed consent to publication for case details was obtained from the patient and the healthy volunteers that participated in the study.

### Case report

CDB, a 55-year-old, right-handed female with previously normal hearing was admitted to our Neurorehabilitation Unit for two consecutive strokes. The first ischemic attack occurred in December 2007 and involved the territories of the middle cerebral artery of the right hemisphere. A week later, a second infarction affected the areas of the middle artery of the left side of the brain.

On admission, the patient was alert and oriented in space and time, but often appeared agitated and perplexed. Neurologic examination showed no sensorimotor deficits, but she complained a complete loss of hearing. Her speech was characterized by a severe dysarthria; she was unable to respond to verbal questions or environmental sounds and startle response to loud noises could not be detected. She could understand and execute complex written instructions and recognize and use gestures for communication. Writing was performed hesitantly with much persuasion and showed graphemic errors and agrammatism. The T_1_-weighted MRI scan showed bilateral lesions of the superior temporal gyrus and its subregions (planum polare, Heschl gyrus, planum temporale), the inferior parietal lobe and angular gyri and the insular cortex. On the right, the lesion extended into the inferior frontal gyrus.

During the first year in our Neurorehabilitation Unit, CDB showed consistent evidence of cortical deafness. Language functions were measured with the Aachen aphasia test (AAT) [11] and revealed that the patient could not understand speech, repeat words and write to dictation. She could recognize and name pictures and match written words with objects. She showed a good visual-spatial memory and the Weigl's test revealed normal executive functions. She presented with buccofacial and constructional apraxia. There were, however, no signs of ideomotor apraxia.

The patient mood progressively improved. She appeared to become aware of her deafness and was distressed by this. The agitation that the patient showed a few days after the lesion, tended to resolve gradually. Occasionally, her relatives reported that she unconsciously manifested some startle reactions with blinking and head-turning in the direction of unexpected sounds.

In two-year follow up we did not find improvements in the clinical features of the patient. She complained complete loss of hearing and she could only communicate through reading and writing. Three years after stroke, CDB showed substantially unchanged clinical and neuropsychological features, confirming a persistent cortical deafness. However, sporadic startle responses were reported.

**Figure 1:**
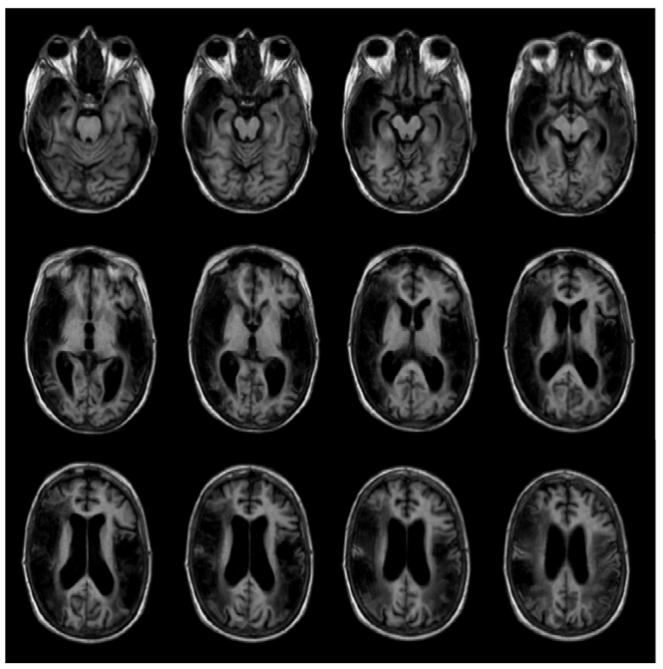
T1-weighted MRI scans of the patient. Consecutive sequences of axial T1-weighted MRI scan of the patient's brain three years after her strokes (right hemisphere is on the left side of the picture). Hypodense lesion massively extends from the bilateral superior temporal gyrus toward the transverse temporal gyrus, the pulvinar, thalamus and insula and the middle temporal gyrus. The right hemisphere was further damaged in the lentiform nucleus, the postcentral gyrus, the inferior frontal gyrus and the inferior parietal lobe.

The MRI scan confirmed the extensive area of infarction in the territory of the bilateral middle cerebral artery. MRI sections of the lesion are shown in Fig. 1. In addition, we compared the voxel-based structural data of the patient with the T1-weighted MRI scans of ten age-matched healthy controls. We used an automated technique of statistical parametric mapping (SPM) to the analysis of gray matter (GM) to detect functional abnormalities that were not accounted for by either visible or unsuspected structural abnormalities. At the corrected threshold of *p*<0.05, the SPM-based morphometric comparison revealed that the patient had a decrease of GM distribution in the bilateral caudate nucleus and the pulvinar thalamus, in addition to the lesional areas delineated with traditional MRI [12]. We planned to perform a functional magnetic resonance (fMRI), but the patient refused the procedure.

### Audiological testing

Audiological examination comprised pure tone audiometry and auditory evoked potentials.

Standard pure-tone air and bone conduction threshold audiometry tested for peripheral hearing impairment using tones of different frequencies with increasing sound pressure intensity, according to the guidelines recommended by the British Society of Audiology [13].

The audiological examination was completed with the auditory brainstem responses (ABRs) and further tests of the central auditory system: middle and long latency auditory evoked potentials (MLAEPs and LLAEPs, respectively). ABRs include short-latency potentials and are associated with the eighth nerve, cochlear nucleus, superior olive, and midbrain. They occur in the first 10 ms following stimulus onset. MLAEPs represent the earliest cortical response to acoustic stimulus and cover the 10–80 ms latency range, including Na, Pa, Nb and Pb components. Finally, N1, P2, and N2 components of LLAEPs occur between 80 and 200 ms after sound onset and are related to auditory system on primary and secondary cortical areas involved in the central auditory process.

Auditory responses were recorded with the patient rested on a bed in an acoustically shielded room. 1-kHz tone bursts were presented monaurally at a frequency of 10 Hz with alternating polarity (condensation and rarefaction clicks). The acoustic stimulus was applied monaurally via TDH-49 earphones. The estimation of auditory threshold was traced by the wave V detection which can be used as a robust indicator that the central nervous system detected an auditory stimulus [14]. The patient exhibited large and reproducible waves V at an intensity of 100 dB. Thus, we adopted the same stimulation intensity to evoke auditory responses. The contralateral ear was masked by 40 dB white noise in all trials. Recordings were derived from the vertex (Cz), referenced to the earlobes (A1 and A2). At least two runs, consisting of 2000 click presentations each, were obtained for all auditory evoked potentials. ABRs were set as follows: 10 ms epochs, bandpass filtering of 150–3000 Hz. Middle-latency response variables were: 80 ms epochs using band-pass of 5–1000 Hz. Late auditory potentials were analysed in 500 ms epochs and band-pass of 0.5–30 Hz.

All auditory signals were inspected visually. All visual analyses were revised by one investigator aware of the patient but blinded to the patient's age and further clinical data. Auditory recordings classified as distorted or insufficient for interpretation were excluded. Individual waveforms were included in a grand average providing a better estimate of peak amplitudes and latencies.

### Event-related potentials (ERPs)

In addition to audiological tests, the later, more cognitive stages of auditory processing were examined in an auditory oddball experiment.

Electroencephalogram (EEG) was recorded from a 32 channel electrode cap, according to the extended 10–20 method of electrode application, referred to linked earlobes with a forehead ground. Additional electrodes were placed below the right eye and at the outer canthus, for bipolar recordings or the electroocular activity (EOG). Stimuli were presented monaurally at 90 dB. Two sinusoid tones of 1000 and 2000 Hz served as frequent and rare tones, respectively. Probability of rare tones was 20% and probability of occurrence of frequent tones was 80%. The interstimulus interval was 1.0–1.3 sec. The experimental session was subdivided into four blocks consisting of 100 stimuli each, separated by 1-min breaks. Data were recorded with a band-pass of 0.15 to 70 Hz and digitized at 1000 Hz (NeuroScan Amplifier, Compumedics Neuroscan) for later off-line analysis. EEG data analysis was performed using EEGLAB 9.0.4, an open source Matlab toolbox. A notch filter was used to eliminate the frequencies centred on 50 Hz. The EEG data were segmented in epochs of 1200 ms including 200 ms pre-stimulus baseline, time-locked to the beginning of the stimulus presentation. Epochs including EEG excursions exceeding ±90 µV were rejected. After averaging, a further digital low-pass filter at 30 Hz was applied.

For assessment of the MMN, we computed the increase in the negativity of the evoked potential in response to the deviants as compared with the response to the standards occurring within a 100–300 ms time window.

P3 was measured relative to the pre-stimulus baseline and was defined as the largest positive component occurring after the N1-P2-N2 complex, within a latency window between 300 and 700 ms after a deviant stimulus was detected.

Auditory oddball ERP signals of the patient were compared with those of ten healthy controls matched for age and gender to gain insight into underlying mechanisms of P3 in cortical deafness. Healthy controls were submitted to the same data collection and analysis conditions as the patient. Topographic maps of P3 distribution were constructed using grand-average voltage information from all scalp electrodes.

Within and between group analysis was performed by the modified t-test described by Crawford and Howell (1998) [15]. Statistical analysis was restricted to P3 findings in terms of peak amplitude at central midline electrodes (Fz, Cz, and Pz), as well as overall brain electrical activity mapping when P3 component exhibited largest amplitude in both groups. Statistical significance was accepted when p<0.05.

## Results

### Audiological testing

Pure tone air and bone conduction audiometry indicated a complete sensorineural hearing loss bilaterally. The patient had no response at output limits of audiometry (110 decibels, dB) in the frequency range of 250–8000 Hz.

**Figure 2:**
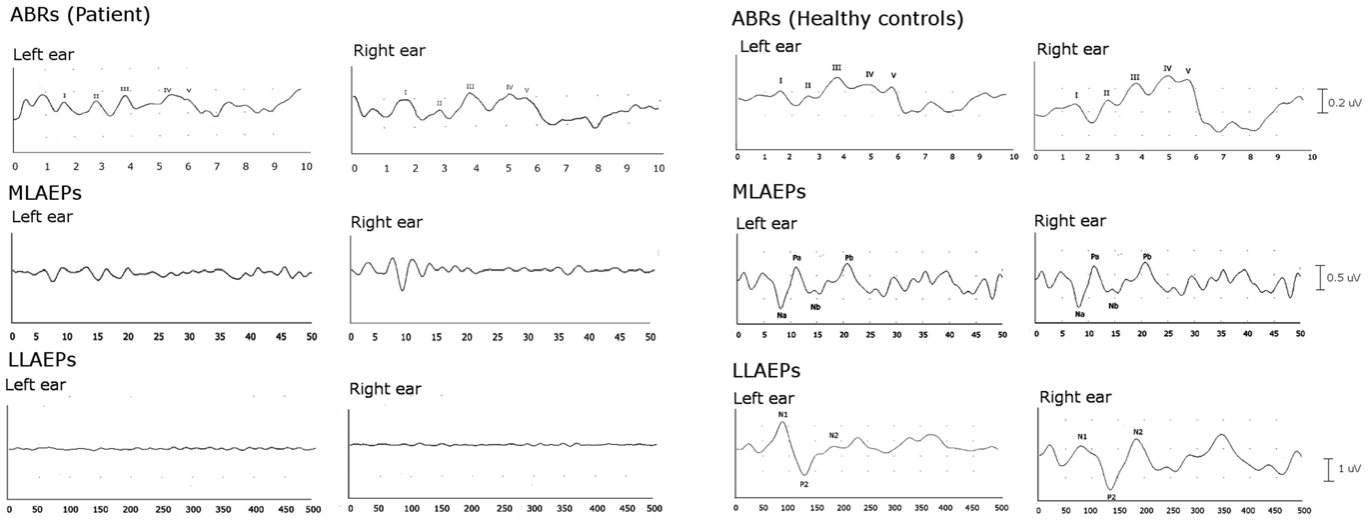
Grand-averaged auditory evoked potentials to sound onsets. Auditory brainstem responses (ABRs; top), middle-latency auditory evoked potentials (MLAEPs; middle), and long-latency auditory evoked potentials (LLAEPs; bottom) recorded by routine clinical protocols in the patient with cortical deafness (left) and in the control group (right). Note the integrity of brainstem auditory responses and the absence of bilateral middle- and long-latency evoked potentials, characterizing cortical deafness.

Figure 2 shows the grand-averaged waveforms of auditory evoked potentials for the two runs of stimulation. Brainstem auditory potentials of either ear evoked essentially normal responses suggesting the integrity of the auditory nerves and pathways up to the inferior colliculi of both sides.

Conversely, middle latency auditory responses (Na, Pa, Nb and Pb) and long latency potentials with N1, P2 and N2 could not be identified reliably.

### Event-related potentials (ERPs)

**Figure 3:**
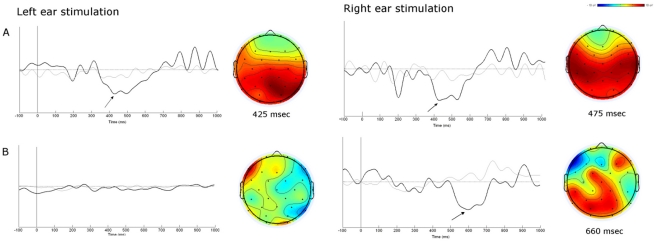
Grand-averaged event related potentials (ERPs) elicited by classic oddball at electrode Cz. Grand-averaged ERP waveforms at electrode Cz and thirty-one channel ERP topographical maps recorded in the patient (B) and controls (A). The latency is referred at the time point at which the positive peak between 300 and 700 ms latency is maximum at Cz electrode site. ERP waveform from the patient revealed a significantly higher P3 at the left posterior and central sites in response to right stimulation. No P3 could be recorded after left ear stimuli.

The Mismatch Negativity could not be recorded in response to deviant stimuli. However, robust positive peaks in the latency range of 650–700 ms were clearly seen following the right ear stimulation (Fig. 3B). Contralateral responses were abolished.

Statistical comparison between patient and controls revealed significant differences in P3 scalp distribution. In the within-group analysis, the control group showed a significantly larger right centro-parietal P3 in response to left ear stimulation (p = 0.0001), and a bilateral centro-temporal dominance in response to right ear stimulation (p = 0.01) (Fig. 3A). Differently, the patient exhibited the largest amplitude of P3 at the left posterior and central areas after right ear stimulation (p = 0.0001; p = 0.0001) (Fig. 3B). No P3 could be recorded in response to left ear stimuli. Finally, the between-group analysis showed a significantly lower P3 amplitude over the right fronto-temporal and central regions of the patient compared to controls (*p* = 0.0001; *p* = 0.008, respectively).

## Discussion

In the present study, we reported the rare case of a patient with persistent cortical deafness. This disorder is characterized by bilateral temporo-parietal lesions involving the primary auditory areas and their radiations and implies the complete absence of auditory afferent input at the cortical level. In contrast, several authors have presented evidence of residual capacity of the damaged auditory system to process acoustic stimuli [2], [5], [6].

The goal of the present study was, therefore, a systematic examination of several levels of auditory function using auditory evoked potentials in the attempt to better evaluate the dissociation between perception and higher order processing of acoustic information.

Cortical deafness is generally associated with a damage of central auditory functions, in spite of normal peripheral hearing. Accordingly, our patient showed normal ABRs, expression of the integrity of the inner ear and cochlear nerve, as well as the auditory pathways within the brainstem. However, middle-latency responses were consistently absent to both right and left ear stimulation. The neural origin of MLAEPs is still a subject of controversy. Although some authors support the hypothesis of a subcortical involvement in the potential generation, it is generally assumed that MLAEPs arise from the medial geniculate and the supratemporal plane comprising the primary auditory area (A1), and the surrounding region of the superior temporal gyrus and the frontal and parietal operculum [16], [17]. Thus, in our patient the extent of the brain damage and the absence of MLAEP waves provide evidence of a cortical origin of the mid-latency potentials.

Concerning the long-latency auditory potentials, recent neuroimaging data have suggested that abnormalities in the P1, N1 and P2 components reflect lesions extended into the multi-modal areas of the inferior parietal lobule. This area appears to exert a critical modulatory influence over LLAEP generators outside of the superior temporal plane [18]. The absence of LLAEPs in our patient does not necessarily reflect a damage to primary auditory cortex, but also a damage to adjacent posterior areas.

Finally, we recorded two main components of ERPs: the Mismatch Negativity and the P3. Although we supposed to observe an absence of endogenous potentials, we found some unexpected results.

MMN could not be recorded in response to deviant sounds. Generally, the MMN appears in response to changes in sound stimulation and is known to reflect a relatively automatic comparison of incoming sounds to auditory cortex sensory-memory representations of the preceding repetitive stimuli [19]. Some authors have suggested that these representations might be explained by the transient adaptation of feature-specific neurons within the anterior and posterior parts of the primary auditory cortex that regulate the access to the conscious perception of sound [20], [21]. In fact, numerous brain imaging studies on preattentive auditory deviance detection have demonstrated an initial contribution of the primary auditory cortex followed by the activation of the posterior superior temporal gyrus and the lateral planum temporale. These areas might be involved in the withdrawal of the details of the acoustic change [22], [23]. Through the connections of the arcuate and superior longitudinal fascicle, the superior temporal gyrus is in communication with the inferior frontal gyrus. The activation of this region of the mid-ventrolateral prefrontal cortex might indicate a higher cognitive processing related to the judgement of sufficient novelty of auditory stimulus to require attentional resources [24]. The brain damage of CDB extended to the primary auditory cortex, the superior temporal gyrus, planum temporale and inferior frontal gyrus and could hamper sounds to access consciousness.

In contrast with the absence of the MMN, a reliable positive peak at 660 ms could be clearly detected in response to right ear stimulation. Because of its morphology and topographic distribution, we inferred that such late waveform could represent a P3-like potential.

P3 offers a covert and indirect measure of attentional resource allocation that represents an index of change detection [25]. P3 is related to the activity of associative cortical areas and is sensitive to complex processes around recalled information, stimulus significance, recognized auditory information and memory context updating [26], [27]. The sources of P3 are believed to be located in heteromodal areas of the fronto-parietal cortex and their activation might reflect an attention switch to an environmental change encoded by the cerebral process generating the MMN [28], [29]. The bilateral supramarginal gyrus, frontal operculum and insula seem to be mainly involved in the network for saliency detection in auditory modality [30]. However, some authors have demonstrated an asymmetrical cortical activation of P3 by using unilateral auditory stimulation. Among others, Gilmore and colleagues (2009) argued that in normal condition the right hemisphere is more prominently engaged during working memory and updating processes underlying P3 [31]. Accordingly, our healthy controls exhibited a right lateralized potential in response to left ear stimulation, and a bilateral distribution of ERPs to right ear stimulation. This denotes a more marked right side activation of P3 wave (Fig. 3A). In contrast, the patient showed robust P3-like components over the left posterior areas and a significantly lower distribution of the potentials over the right fronto-temporal and central areas in response to right ear stimulation. The left ear stimulation could not evoke any detectable responses. (Fig. 3B).

Depth recordings and lesion studies have implicated the frontal, temporal and inferior parietal lobes in the generation of the auditory P3 [32]. The interaction of these structures seems to play a key role in the auditory perceptual awareness, and the right hemisphere seems to be mainly engaged during working memory updating processes [31], [33]. Thus, the marked right fronto-parietal dysfunction of our patient, in particular the damage of the right inferior frontal gyrus and the inferior parietal lobe, might partially explain the dissociation between detection and perception of sounds. Several authors have demonstrated that ERPs can be elicited even when stimuli are presented outside conscious awareness [34], [35]. Bernat et al. (2001) offer evidence that subliminal stimuli can evoke consistent P3 waves. They speculated that P3 could represent a link between unconscious and conscious awareness in the context updating processes [35]. In our patient the generation of P3-like potentials implied that deviant stimuli were selectively processed bypassing networks involved in conscious perception. Schönwiesner et al. (2007) and Pandya (1995) hypothesized that association areas in and adjacent to the auditory parabelt might form an independent circuit from thalamo-cortical projections in the auditory system [26], [36]. These alternate pathways could be preserved in our patient and responsible for the generation of P3-like potentials.

In addition, the lack of awareness of auditory stimuli might be further aggravated by the bilateral functional abnormalities of the pulvinar. In fact, the auditory association area seems to be preferentially related to the pulvinar complex [36]. The thalamic pulvinar nucleus plays an important role in the coupling of sensory and attentional functions. As demonstrated by Hugdahl et al. (1991), a lesion of this structure might imply an auditory neglect that further affected the conscious awareness of sounds of CDB [37].

As a final consideration, our findings seem to be in contrast with the hypothesis of a hierarchical information processing of auditory stimuli. A large body of physiological and functional data suggests that processing of auditory information is implemented in a hierarchical manner [38]. Lower levels are considered responsible for extracting basic spectro-temporal features of the auditory signal. Higher cortical areas are involved in a higher level of information processing, such as abstraction, perception, reasoning, and learning. They are characterized by at least four different, hierarchically organized processing levels, each containing several segregated sub-regions: the primary areas that receive their input from the thalamus; surrounding lateral and medial “belt” areas, that receive input from primary areas; a parabelt area on the dorsal plane of the superior temporal gyrus; and higher-level areas in the superior temporal sulcus and the frontal lobe. From that, the higher level integrative functions evolved from and are dependent on the integrity of lower-level structures. In other words, simple processing operations are necessary prerequisites for more complex operations [39], [40].

Our results are rather compatible with a growing body of literature demonstrating another line of thought, based on the reverse hierarchy theory (RHT) [41]. The reverse hierarchy theory provides a representational hierarchy to describe the interaction between sensory input and top–down processes to guide plasticity in primary sensory areas [42]–[44]. RHT asserts that neural circuits mediating a certain percept can be modified starting at the highest representational level and progressing to lower-levels in search of more refined high resolution information to optimize perception. RHT may be a plausible explanation for top–down influences on cortical levels of sensory processing [45]. This might further clarify the presence of higher order auditory cortical responses, even when more automatic components are lacking.

In conclusion, the paradoxical partial preservation of P3-like potentials in a patient with persistent cortical deafness suggests the integrity of independent neural circuits necessary to process auditory stimuli even in the absence of conscious awareness. Furthermore, electrophysiological techniques combined with functional neuroimaging could be of primary importance in demonstrating the activation of neural substrates underlying deaf-hearing.
